# Trends in total fat and fatty acid intakes and chronic health conditions in Korean adults over 2007–2015

**DOI:** 10.1017/S1368980018003701

**Published:** 2019-02-15

**Authors:** SuJin Song, Jae Eun Shim, Won O Song

**Affiliations:** 1 Department of Food and Nutrition, Hannam University, Daejeon, South Korea; 2 Department of Food and Nutrition, Daejeon University, 62 Daehak-ro, Dong-gu, Daejeon 34520, South Korea; 3 Department of Food Science and Human Nutrition, Michigan State University, East Lansing, MI, USA

**Keywords:** Total fat, Fatty acids, Obesity, Hypercholesterolaemia, Korean adults

## Abstract

**Objective:**

To investigate trends in total fat and fatty acid intakes and chronic health conditions among Korean adults over nine years between 2007 and 2015.

**Design:**

Cross-sectional, observational study using a stratified, multistage probability sampling design at a national level. Intakes of total fat and fatty acids were estimated from 24 h dietary recalls by sex and age groups. Trends of total fat and fatty acid intakes were determined by multiple linear regression after adjusting for covariates. Trends in age-standardized prevalence of obesity, hypercholesterolaemia and hypertriacylglycerolaemia were examined by sex.

**Setting:**

Korea.

**Participants:**

Population data of 47749 healthy adults (aged ≥19 years) derived from the Korea National Health and Nutrition Examination Survey between 2007 and 2015.

**Results:**

Over the survey period, daily intakes of energy and total, saturated, monounsaturated, polyunsaturated, *n*-3 and *n*-6 fats (grams and percentage of energy (%E)) increased steadily. In all sex and age groups, significant increases were seen in SFA intake from 9·9 g (4·7 %E) to 12·0 g (5·3 %E) and in MUFA intake from 9·9 g (4·6 %E) to 13·3 g (5·8 %E). The prevalence of hypercholesterolaemia increased from 10·7 to 17·9 % over the same period.

**Conclusions:**

In Korean adults, total fat, SFA and other fatty acids have been increasing along with the prevalence of hypercholesterolaemia. This information can help set adequate macronutrient and fatty acid distribution ranges in developing population-specific preventive strategies against diet-related illness.

For the past few decades, non-communicable diseases (NCD) such as CHD, stroke, diabetes and cancers have been leading causes of mortality and disability worldwide. Elevated LDL-cholesterol, high blood pressure, being overweight or obese, and smoking have been established as modifiable risk factors for CHD^(^ ^1^ ^)^. Several classical reports supported that total fat and saturated fat intakes significantly increase the blood LDL-cholesterol level^(^ ^2^ ^)^. Therefore, dietary fat, particularly saturated fat, has been considered a potent dietary risk factor for CHD.

Recently systematic reviews of randomized controlled trials and prospective cohort studies have reported that saturated fat intake is not associated with CVD^(^ ^3^ ^–^ ^5^ ^)^. However, the randomized controlled trials and prospective cohort studies included in those systematic reviews did not account for the nutrients that replaced the fats or saturated fats^(^ ^6^ ^–^ ^8^ ^)^. Several other studies have pointed out that the reduction of dietary fats is often accomplished by replacing of saturated fats with carbohydrates, especially sugars, which results in obesity and other related diseases^(^ ^9^ ^)^. A number of other studies have indicated that dietary saturated fat, in contrast to complex carbohydrates and *cis*-unsaturated fatty acids, raises LDL-cholesterol and, in turn, the risk of CHD^(^ ^10^ ^,^ ^11^ ^)^. Thus, the impact of modifying one’s diet with a focus on fat composition still needs to be examined.

The worldwide increase in diet-related NCD has been accompanied by the increased consumption of more energy-dense and sweeter foods^(^ ^12^ ^)^. The increased consumption of animal-source foods, main sources of saturated fat and cholesterol, has also played an important role in the changes of dietary patterns^(^ ^13^ ^)^. Efforts to improve dietary intakes in certain sociodemographic subgroups of Western countries have somewhat improved diet-related health problems, while the situation has persisted in other subgroups of such countries as well as in other parts of developing countries, where a double burden of inadequate nutrient intake and high consumption of energy and fat exists with diet-related NCD^(^ ^14^ ^)^.

The current disparity in eating patterns and health statuses among various race/ethnicity groups, regions and countries suggests the need for differentiated approaches that are specific to different diet and health contexts. In Korea, heart disease is the second-leading cause of mortality. Scientists and policy makers in Korea have debated the ideal macronutrient intake levels to prevent diet-related illness, but estimations of population-level consumption of different types of fat have not been possible thus far due to lack of a comprehensive fatty acid food composition database. Recently, a fatty acid database was constructed for the preparation of dietary data from the Korea National Health and Nutrition Examination Survey (KNHANES)^(^ ^15^ ^)^, which enabled us to conduct the present study. In the present study, trends in total and types of fat intake, macronutrient contributions to energy and health conditions (i.e. obesity and dyslipidaemia) were investigated among Korean adults. The study used a valid and comprehensive database to document novel evidence for preventive strategies against diet-related illness such as CHD.

## Methods

### Data and participants

The present study was based on data from the fourth (2007–2009), fifth (2010–2012) and sixth (2013–2015) KNHANES. The KNHANES is a national survey designed to assess and monitor the health and nutritional statuses of the Korean population at the national level. It is a cross-sectional and nationally representative survey that is conducted every year by the Korea Centers for Disease Control and Prevention. The survey uses a stratified, multistage probability sampling design and consists of three survey sections: health interview, health examination and nutrition survey. Detailed explanations of the KNHANES are available elsewhere^(^ ^16^ ^)^.

Among the eligible individuals who were aged 19 years or older and had dietary data (*n* 49649), we excluded individuals who were pregnant or breast-feeding (*n* 927) or reported extreme energy intakes, defined in the present study as <1st or >99th percentile of daily energy intake by sex (*n* 973). A total of 47749 participants (20024 men and 27725 women) was included in the final data analyses. The study was conducted according to the guidelines laid out in the Declaration of Helsinki and all procedures involving human subjects were approved by the Korea Centers for Disease Control and Prevention Institutional Review Board. Written informed consent was obtained from all subjects.

### Dietary assessment

Dietary intake data were obtained by a single 24 h dietary recall. The dietary data were collected by trained dietitians at each participant’s home, one week after the completion of the health interview survey and health examination survey. Energy and macronutrient intakes were calculated for each participant using the food composition table published by the Korea Rural Development Administration^(^ ^17^ ^,^ ^18^ ^)^. Energy and macronutrient intakes were presented as absolute amounts in grams as well as proportions (i.e. percentage of total energy intake (%E)). Fatty acid intakes in the sixth KNHANES were calculated using a new database which was developed in 2014^(^ ^15^ ^)^. This database contains the fatty acid contents of 5144 Korean foods items used in the KNHANES data. Analytical values of fatty acid contents in foods were compiled from domestic and foreign sources based on the evaluation of data quality. In cases of missing values, the fatty acid contents were calculated or imputed based on the analytical values of similar foods. The process of developing the database is described in detail elsewhere^(^ ^15^ ^)^. At the time of the fourth and fifth KNHANES surveys, fatty acid composition tables were not available. Thus, fatty acid intakes in the fourth and fifth KNHANES were calculated using the fatty acid contents per 100 g of foods which were replaced with the sixth KNHANES data. For foods which did not appear in the sixth KNHANES, the fatty acid contents were replaced with calculated or imputed values using the fatty acid contents of similar foods in the sixth KNHANES data or in the US Department of Agriculture fatty acid database^(^ ^19^ ^)^. Regarding mixed foods, the fatty acid contents of the main food source for total fat were applied.

### Chronic health conditions

Information on chronic health conditions was obtained from the report of Korea Health Statistics 2015 published by the Korea Centers for Disease Control and Prevention^(^ ^20^ ^)^. The chronic health conditions of interest included obesity, hypercholesterolaemia and hypertriacylglycerolaemia. Obesity was defined as BMI ≥25·0 kg/m^2^ among adults aged ≥19 years, according to the WHO Asia-Pacific region criteria^(^ ^21^ ^)^. Hypercholesterolaemia and hypertriacylglycerolaemia were diagnosed based on the National Cholesterol Education Program Adult Treatment Panel III criteria^(^ ^22^ ^)^. Hypercholesterolaemia was defined as total cholesterol ≥240 mg/dl or the use of medication among adults aged ≥30 years. Hypertriacylglycerolaemia was defined as fasting TAG ≥200 mg/dl among adults aged ≥30 years. To adjust for differences in the age structure of each year survey, the age-standardized prevalence of chronic health conditions was presented using sex- and age-specific structures of the estimated population based on the 2005 Korea Census from 2007 through 2015 by sex.

### Statistical analyses

All statistical analyses were conducted using the statistical software package SAS version 9.4. All analyses accounted for the complex sampling design effect and used appropriate sampling weights, using the PROC SURVEY in the SAS program to produce estimates of the entire Korean adult population from the representative survey sample. Dietary variables were expressed as means with se by sex and age groups. Linear trends in intakes of total fat and fatty acids across the survey periods were compared using the generalized linear model after adjusting for sex, age, region and household income, where applicable.

## Results

### Trends in intakes of energy and macronutrients

Total energy and macronutrient intakes from 2007 through 2015 are summarized by sex in Table 1. In both sexes, from 2007 to 2015, the total energy intake and percentage of energy from fat increased, whereas the percentage of energy from carbohydrate decreased from 2007 through 2015.

**Table 1:** Energy and macronutrient intakes in Korean adults from 2007 to 2015 by sex*. Data from the fourth (2007–2009), fifth (2010–2012) and sixth (2013–2015) Korea National Health and Nutrition Examination Survey

	2007–2009		2010–2012		2013–2015		
	Mean	se	Mean	se	Mean	se	*P* trend†
Total (*n* 47749)							
Total energy (kJ)	7991·4	39·7	8536·6	42·7	8643·7	40·6	<0·0001
Total energy (kcal)	1910·0	9·5	2040·3	10·2	2065·9	9·7	<0·0001
Carbohydrate (%E)	67·7	0·2	66·7	0·2	65·6	0·1	<0·0001
Protein (%E)	14·8	0·1	14·8	0·1	14·6	0·1	0·0856
Fat (%E)	17·6	0·1	18·6	0·1	19·8	0·1	<0·0001
Male (*n* 20024)							
Total energy (kJ)	9392·2	59·4	10074·7	61·1	10109·8	59·8	<0·0001
Total energy (kcal)	2244·8	14·2	2407·9	14·6	2416·3	14·3	<0·0001
Carbohydrate (%E)	65·9	0·2	65·0	0·2	64·2	0·2	<0·0001
Protein (%E)	15·4	0·1	15·4	0·1	15·2	0·1	0·0649
Fat (%E)	18·7	0·2	19·6	0·2	20·6	0·1	<0·0001
Female (*n* 27725)							
Total energy (kJ)	6569·7	33·1	6978·9	36·8	7152·1	35·1	<0·0001
Total energy (kcal)	1570·2	7·9	1668·0	8·8	1709·4	8·4	<0·0001
Carbohydrate (%E)	69·4	0·2	68·3	0·2	67·0	0·2	<0·0001
Protein (%E)	14·1	0·1	14·2	0·1	14·1	0·1	0·4340
Fat (%E)	16·5	0·1	17·5	0·1	19·0	0·1	<0·0001

%E, percentage of energy.

* All statistical analyses accounted for the complex sampling design effect and appropriate sampling weights.

† Unadjusted.

### Trends in intakes of total fat and fatty acids

Trends in intakes of total fat and fatty acids across survey periods are presented by sex and age groups in Tables 2, 3 and 4. In all sex and age groups, total fat intake (g and %E) increased significantly from 2007 to 2015 (*P*<0·05). In addition, all sex and age groups showed significant increases in SFA intake: from 11·8 g (5·0 %E) in 2007–2009 to 14·0 g (5·5 %E) in 2013–2015 among men and from 7·8 g (4·5 %) in 2007–2009 to 10·0 g (5·2 %E) in 2013–2015 among women. MUFA intake increased from 9·9 to 13·3 g (*P*<0·0001) from 2007 through 2015, as did the proportion of energy from MUFA from 4·6 to 5·8 %E (*P*<0·0001). PUFA intake (g as well as %E) also significantly increased in all sex and age groups except for young adults aged 19–29 years. The intake of *n*-6 fatty acids increased in most sex and age groups, while the intake of *n*-3 fatty acids increased significantly only in adults aged ≥50 years.

**Table 2:** Total fat intake (grams and percentage of energy (%E)) in Korean adults from 2007 to 2015 by sex and age group*. Data from the fourth (2007–2009), fifth (2010–2012) and sixth (2013–2015) Korea National Health and Nutrition Examination Survey

	Total fat (g)							Total fat (%E)						
	2007–2009		2010–2012		2013–2015			2007–2009		2010–2012		2013–2015		
	Mean	se	Mean	se	Mean	se	*P* trend†	Mean	se	Mean	se	Mean	se	*P* trend†
Total	33·7	0·3	39·6	0·3	42·2	0·3	<0·0001	16·0	0·1	17·3	0·1	18·7	0·1	<0·0001
Sex														
Male	40·2	0·5	47·2	0·5	49·0	0·5	<0·0001	16·9	0·1	18·1	0·1	19·2	0·1	<0·0001
Female	26·8	0·3	31·6	0·3	35·1	0·3	<0·0001	15·2	0·1	16·5	0·1	18·2	0·1	<0·0001
Age														
19–29 years	48·9	0·9	57·9	1·1	58·7	0·9	<0·0001	22·6	0·2	24·4	0·3	25·0	0·2	<0·0001
30–49 years	41·5	0·5	47·8	0·6	50·8	0·5	<0·0001	18·7	0·1	20·1	0·1	21·8	0·2	<0·0001
50–64 years	30·8	0·5	35·3	0·5	39·3	0·6	<0·0001	14·6	0·2	15·8	0·2	17·4	0·2	<0·0001
≥65 years	21·0	0·4	23·2	0·4	25·2	0·4	<0·0001	11·6	0·2	11·8	0·1	13·0	0·2	<0·0001
Sex×age														
Male, 19–29 years	57·6	1·5	67·3	1·7	67·9	1·5	<0·0001	22·9	0·3	24·4	0·4	25·3	0·3	<0·0001
Male, 30–49 years	50·4	0·7	57·7	0·9	59·3	0·8	<0·0001	19·8	0·2	20·9	0·2	22·2	0·2	<0·0001
Male, 50–64 years	36·9	0·7	42·1	0·8	45·4	0·9	<0·0001	15·6	0·2	16·8	0·2	17·9	0·2	<0·0001
Male, ≥65 years	25·1	0·7	29·0	0·6	30·2	0·6	<0·0001	12·4	0·2	13·1	0·2	14·0	0·2	<0·0001
Female, 19–29 years	40·2	0·8	48·4	1·1	49·6	1·0	<0·0001	22·3	0·3	24·4	0·4	24·6	0·3	<0·0001
Female, 30–49 years	32·6	0·4	37·9	0·5	42·3	0·6	<0·0001	17·7	0·2	19·2	0·2	21·4	0·2	<0·0001
Female, 50–64 years	24·8	0·5	28·6	0·5	33·1	0·5	<0·0001	13·6	0·2	14·8	0·2	16·9	0·2	<0·0001
Female, ≥65 years	16·7	0·4	17·6	0·4	20·1	0·4	<0·0001	10·6	0·2	10·6	0·2	12·0	0·2	<0·0001

* All statistical analyses accounted for the complex sampling design effect and appropriate sampling weights.

† Linear trends in total fat intake across the survey periods were compared using the generalized linear model after adjustment for sex, age, region and household income, where applicable.

**Table 3:** SFA intake (grams and percentage of energy (%E)) in Korean adults from 2007 to 2015 by sex and age group*. Data from the fourth (2007–2009), fifth (2010–2012) and sixth (2013–2015) Korea National Health and Nutrition Examination Survey

	SFA (g)							SFA (%E)						
	2007–2009		2010–2012		2013–2015			2007–2009		2010–2012		2013–2015		
	Mean	se	Mean	se	Mean	se	*P* trend†	Mean	se	Mean	se	Mean	se	*P* trend†
Total	9·9	0·1	11·7	0·1	12·0	0·1	<0·0001	4·7	0·03	5·1	0·04	5·3	0·03	<0·0001
Sex														
Male	11·8	0·2	14·0	0·2	14·0	0·2	<0·0001	5·0	0·05	5·4	0·05	5·5	0·05	<0·0001
Female	7·8	0·1	9·4	0·1	10·0	0·1	<0·0001	4·5	0·04	4·9	0·04	5·2	0·04	<0·0001
Age														
19–29 years	14·9	0·3	17·7	0·4	17·7	0·3	<0·0001	6·9	0·1	7·5	0·1	7·6	0·1	<0·0001
30–49 years	12·3	0·2	14·3	0·2	14·7	0·2	<0·0001	5·6	0·1	6·0	0·1	6·3	0·1	<0·0001
50–64 years	8·9	0·1	10·2	0·2	10·8	0·2	<0·0001	4·2	0·1	4·6	0·1	4·8	0·1	<0·0001
≥65 years	6·1	0·1	6·8	0·1	7·0	0·1	<0·0001	3·3	0·1	3·5	0·1	3·6	0·1	0·0002
Sex×age														
Male, 19–29 years	17·7	0·5	20·4	0·6	20·3	0·5	0·0006	7·0	0·1	7·4	0·2	7·6	0·1	0·0016
Male, 30–49 years	14·9	0·2	17·3	0·3	17·3	0·3	<0·0001	5·9	0·1	6·3	0·1	6·5	0·1	<0·0001
Male, 50–64 years	10·7	0·2	12·3	0·2	12·7	0·3	<0·0001	4·5	0·1	4·9	0·1	5·0	0·1	<0·0001
Male, ≥65 years	7·3	0·2	8·5	0·2	8·3	0·2	0·0011	3·6	0·1	3·9	0·1	3·9	0·1	0·0227
Female, 19–29 years	12·2	0·3	15·1	0·4	15·1	0·3	<0·0001	6·8	0·1	7·6	0·1	7·5	0·1	0·0002
Female, 30–49 years	9·7	0·1	11·3	0·2	12·2	0·2	<0·0001	5·2	0·1	5·8	0·1	6·2	0·1	<0·0001
Female, 50–64 years	7·1	0·1	8·1	0·1	8·9	0·2	<0·0001	3·9	0·1	4·3	0·1	4·6	0·1	<0·0001
Female, ≥65 years	4·7	0·1	5·1	0·1	5·5	0·1	<0·0001	3·1	0·1	3·1	0·1	3·3	0·1	0·0004

* All statistical analyses accounted for the complex sampling design effect and appropriate sampling weights.

† Linear trends in SFA intake across the survey periods were compared using the generalized linear model after adjustment for sex, age, region and household income, where applicable.

**Table 4:** Intakes of unsaturated fatty acids (grams and percentage of energy (%E)) in Korean adults from 2007 to 2015 by sex and age group*. Data from the fourth (2007–2009), fifth (2010–2012) and sixth (2013–2015) Korea National Health and Nutrition Examination Survey

	MUFA (g)							MUFA (%E)						
	2007–2009		2010–2012		2013–2015			2007–2009		2010–2012		2013–2015		
	Mean	se	Mean	se	Mean	se	*P* trend†	Mean	se	Mean	se	Mean	se	*P* trend†
Total	9·9	0·1	12·0	0·1	13·3	0·1	<0·0001	4·6	0·04	5·1	0·04	5·8	0·04	<0·0001
Sex														
Male	11·9	0·2	14·4	0·2	15·5	0·2	<0·0001	4·9	0·1	5·4	0·1	6·0	0·1	<0·0001
Female	7·7	0·1	9·4	0·1	10·9	0·1	<0·0001	4·3	0·04	4·8	0·04	5·6	0·04	<0·0001
Age														
19–29 years	15·3	0·3	18·5	0·4	19·1	0·4	<0·0001	7·0	0·1	7·7	0·1	8·1	0·1	<0·0001
30–49 years	12·5	0·2	14·7	0·2	16·3	0·2	<0·0001	5·6	0·1	6·1	0·1	6·9	0·1	<0·0001
50–64 years	8·8	0·2	10·4	0·2	12·1	0·2	<0·0001	4·1	0·1	4·6	0·1	5·3	0·1	<0·0001
≥65 years	5·8	0·1	6·5	0·1	7·4	0·1	<0·0001	3·1	0·1	3·3	0·1	3·8	0·1	<0·0001
Sex×age														
Male, 19–29 years	18·2	0·5	21·6	0·6	22·4	0·6	<0·0001	7·2	0·1	7·8	0·2	8·3	0·2	<0·0001
Male, 30–49 years	15·3	0·3	18·0	0·4	19·1	0·3	<0·0001	5·9	0·1	6·4	0·1	7·1	0·1	<0·0001
Male, 50–64 years	10·7	0·3	12·5	0·3	14·2	0·3	<0·0001	4·5	0·1	4·9	0·1	5·5	0·1	<0·0001
Male, ≥65 years	7·0	0·2	8·3	0·2	9·0	0·2	<0·0001	3·4	0·1	3·7	0·1	4·1	0·1	<0·0001
Female, 19–29 years	12·4	0·3	15·4	0·4	15·8	0·4	<0·0001	6·8	0·1	7·7	0·1	7·8	0·1	<0·0001
Female, 30–49 years	9·7	0·1	11·4	0·2	13·5	0·2	<0·0001	5·2	0·1	5·8	0·1	6·8	0·1	<0·0001
Female, 50–64 years	6·9	0·2	8·2	0·2	10·0	0·2	<0·0001	3·8	0·1	4·2	0·1	5·1	0·1	<0·0001
Female, ≥65 years	4·4	0·1	4·8	0·1	5·7	0·1	<0·0001	2·8	0·1	2·8	0·1	3·4	0·1	<0·0001

	PUFA (g)							PUFA (%E)						
	2007–2009		2010–2012		2013–2015			2007–2009		2010–2012		2013–2015		
	Mean	se	Mean	se	Mean	se	*P* trend†	Mean	se	Mean	se	Mean	se	*P* trend†
Total	8·7	0·1	9·8	0·1	10·7	0·1	<0·0001	4·2	0·03	4·3	0·03	4·7	0·03	<0·0001
Sex														
Male	10·3	0·1	11·6	0·1	12·2	0·1	<0·0001	4·4	0·04	4·5	0·04	4·8	0·04	<0·0001
Female	7·1	0·1	7·9	0·1	9·0	0·1	<0·0001	4·0	0·04	4·1	0·04	4·7	0·04	<0·0001
Age														
19–29 years	12·2	0·3	13·7	0·3	13·9	0·3	<0·0001	5·7	0·09	5·8	0·09	5·9	0·09	0·0649
30–49 years	10·4	0·1	11·6	0·2	12·4	0·2	<0·0001	4·7	0·04	4·9	0·05	5·3	0·05	<0·0001
50–64 years	8·1	0·1	9·0	0·1	10·5	0·2	<0·0001	3·9	0·05	4·0	0·05	4·7	0·05	<0·0001
≥65 years	5·7	0·1	6·1	0·1	6·9	0·1	<0·0001	3·1	0·05	3·1	0·04	3·6	0·05	<0·0001
Sex×age														
Male, 19–29 years	14·2	0·4	16·2	0·5	16·1	0·4	0·0018	5·7	0·11	5·9	0·13	6·0	0·12	0·0734
Male, 30–49 years	12·7	0·2	13·9	0·2	14·5	0·2	<0·0001	5·0	0·06	5·1	0·06	5·4	0·06	<0·0001
Male, 50–64 years	9·5	0·2	10·5	0·2	11·8	0·2	<0·0001	4·1	0·07	4·2	0·06	4·7	0·07	<0·0001
Male, ≥65 years	6·6	0·2	7·3	0·2	8·1	0·2	<0·0001	3·3	0·07	3·3	0·06	3·8	0·07	<0·0001
Female, 19–29 years	10·2	0·3	11·2	0·3	11·8	0·3	0·0003	5·7	0·12	5·7	0·11	5·8	0·12	0·3690
Female, 30–49 years	8·2	0·1	9·3	0·2	10·4	0·2	<0·0001	4·5	0·05	4·7	0·06	5·2	0·06	<0·0001
Female, 50–64 years	6·7	0·1	7·5	0·1	9·2	0·2	<0·0001	3·7	0·06	3·9	0·06	4·7	0·07	<0·0001
Female, ≥65 years	4·6	0·1	4·8	0·1	5·6	0·1	<0·0001	3·0	0·06	2·9	0·05	3·4	0·06	<0·0001

	*n*-3 FA (g)							*n*-3 FA (%E)						
	2007–2009		2010–2012		2013–2015			2007–2009		2010–2012		2013–2015		
	Mean	se	Mean	se	Mean	se	*P* trend†	Mean	se	Mean	se	Mean	se	*P* trend†
Total	1·40	0·02	1·52	0·02	1·57	0·02	<0·0001	0·68	0·01	0·68	0·01	0·72	0·01	0·0001
Sex														
Male	1·65	0·03	1·78	0·03	1·79	0·03	0·0003	0·71	0·01	0·71	0·01	0·72	0·01	0·2046
Female	1·14	0·02	1·26	0·02	1·35	0·02	<0·0001	0·65	0·01	0·66	0·01	0·71	0·01	<0·0001
Age														
19–29 years	1·55	0·04	1·69	0·04	1·66	0·04	0·066	0·72	0·01	0·73	0·01	0·71	0·01	0·5538
30–49 years	1·56	0·02	1·71	0·03	1·67	0·03	0·0025	0·72	0·01	0·73	0·01	0·73	0·01	0·3487
50–64 years	1·41	0·03	1·52	0·03	1·72	0·04	<0·0001	0·69	0·01	0·69	0·01	0·78	0·01	<0·0001
≥65 years	1·05	0·03	1·09	0·03	1·23	0·03	<0·0001	0·59	0·01	0·56	0·01	0·64	0·01	0·0017
Sex×age														
Male, 19–29 years	1·80	0·07	1·98	0·07	1·92	0·06	0·2311	0·72	0·02	0·73	0·02	0·72	0·02	0·9339
Male, 30–49 years	1·86	0·04	1·99	0·04	1·92	0·04	0·2744	0·74	0·01	0·73	0·01	0·73	0·01	0·4865
Male, 50–64 years	1·64	0·04	1·76	0·05	1·89	0·05	0·0004	0·71	0·02	0·72	0·02	0·76	0·02	0·0447
Male, ≥65 years	1·22	0·04	1·31	0·04	1·44	0·04	0·0003	0·61	0·02	0·60	0·02	0·68	0·02	0·0037
Female, 19–29 years	1·30	0·04	1·40	0·04	1·41	0·05	0·0834	0·72	0·02	0·72	0·02	0·69	0·02	0·2575
Female, 30–49 years	1·25	0·02	1·43	0·03	1·42	0·03	<0·0001	0·69	0·01	0·72	0·01	0·73	0·01	0·0224
Female, 50–64 years	1·18	0·03	1·28	0·03	1·55	0·04	<0·0001	0·66	0·02	0·66	0·01	0·80	0·02	<0·0001
Female, ≥65 years	0·87	0·03	0·88	0·03	1·02	0·03	0·0006	0·56	0·02	0·52	0·01	0·61	0·02	0·0299

	*n*-6 FA (g)							*n*-6 FA (%E)						
	2007–2009		2010–2012		2013–2015			2007–2009		2010–2012		2013–2015		
	Mean	se	Mean	se	Mean	se	*P* trend†	Mean	se	Mean	se	Mean	se	*P* trend†
Total	7·4	0·1	8·4	0·1	9·2	0·1	<0·0001	3·5	0·03	3·7	0·03	4·1	0·03	<0·0001
Sex														
Male	8·8	0·1	9·9	0·1	10·5	0·1	<0·0001	3·7	0·04	3·8	0·04	4·1	0·04	<0·0001
Female	6·0	0·1	6·7	0·1	7·7	0·1	<0·0001	3·4	0·03	3·5	0·03	4·0	0·03	<0·0001
Age														
19–29 years	10·7	0·2	12·2	0·3	12·4	0·2	<0·0001	5·0	0·08	5·1	0·08	5·2	0·08	0·0235
30–49 years	9·0	0·1	10·0	0·1	10·8	0·1	<0·0001	4·0	0·04	4·2	0·04	4·6	0·04	<0·0001
50–64 years	6·8	0·1	7·6	0·1	8·8	0·1	<0·0001	3·2	0·04	3·4	0·04	3·9	0·05	<0·0001
≥65 years	4·6	0·1	5·0	0·1	5·7	0·1	<0·0001	2·6	0·04	2·5	0·04	2·9	0·05	<0·0001
Sex×age														
Male, 19–29 years	12·5	0·4	14·4	0·4	14·4	0·4	0·0008	5·0	0·10	5·2	0·12	5·3	0·11	0·0392
Male, 30–49 years	10·9	0·2	12·1	0·2	12·6	0·2	<0·0001	4·3	0·05	4·4	0·06	4·7	0·06	<0·0001
Male, 50–64 years	7·9	0·2	8·8	0·2	10·0	0·2	<0·0001	3·4	0·06	3·5	0·06	4·0	0·06	<0·0001
Male, ≥65 years	5·4	0·2	6·1	0·1	6·8	0·2	<0·0001	2·7	0·06	2·8	0·05	3·1	0·06	<0·0001
Female, 19–29 years	9·0	0·2	9·9	0·3	10·5	0·3	<0·0001	5·0	0·11	5·0	0·10	5·1	0·10	0·2105
Female, 30–49 years	7·0	0·1	7·9	0·1	9·0	0·1	<0·0001	3·8	0·04	4·0	0·05	4·5	0·05	<0·0001
Female, 50–64 years	5·6	0·1	6·3	0·1	7·7	0·1	<0·0001	3·1	0·05	3·2	0·05	3·9	0·06	<0·0001
Female, ≥65 years	3·8	0·1	3·9	0·1	4·6	0·1	<0·0001	2·4	0·05	2·3	0·05	2·8	0·05	<0·0001

FA, fatty acids

* All statistical analyses accounted for the complex sampling design effect and appropriate sampling weights.

† Linear trends in intakes of unsaturated fatty acids across the survey periods were compared using the generalized linear model after adjustment for sex, age, region and household income, where applicable.

### Trends in prevalence of obesity and dyslipidaemia

**Fig. 1:**
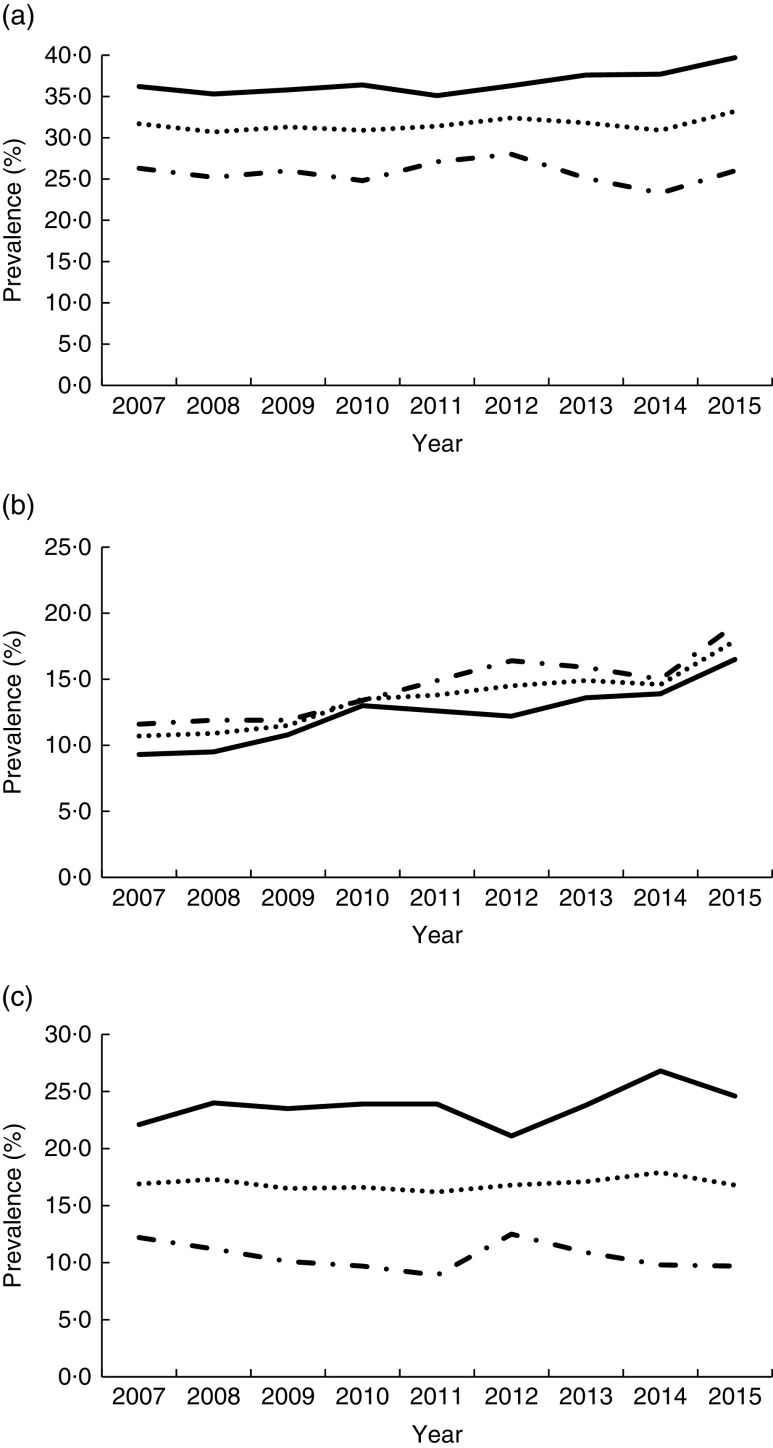
Prevalence of chronic health conditions in Korean adults from 2007 to 2015 by sex (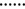, total; 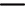, male; 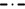, female): (a) obesity; (b) hypercholesterolaemia; (c) hypertriacylglycerolaemia. Data from Korea Health Statistics 2015 report published by the Korea Centers for Disease Control and Prevention^(^ ^20^ ^)^

Trends in prevalence of chronic health conditions from 2007 through 2015 are summarized by sex in Fig. 1. The prevalence of obesity among Korean adults increased from 31·7 % in 2007 to 33·2 % in 2015. This increased rate was prominent only in men, with an increase from 36·2 % in 2007 to 39·7 % in 2015, while a subtle change was seen in women from 26·3 % in 2007 to 26·0 % in 2015. However, the prevalence of hypercholesterolaemia increased gradually from 2007 through 2015 in both men (from 9·3 to 16·5 %) and women (from 11·6 to 19·1 %). The prevalence of hypertriacylglycerolaemia changed with fluctuations from 2007 to 2015 in both men and women.

## Discussion

Popkin suggested early that a sequence of major shifts in human diet and nutritional status in several phases typical in food use corresponded to the changes observed in the prevalence of nutrition-related diseases^(^ ^23^ ^,^ ^24^ ^)^. The author also noted that dietary patterns have converged towards diets high in saturated fat, sugar and refined foods of low dietary fibre, leading to the increased prevalence of obesity and the related chronic degenerative diseases^(^ ^23^ ^)^.

Early studies on SFA and cardiovascular health indicated that dietary SFA and cholesterol intakes led to increases in CHD and hypercholesterolaemia^(^ ^2^ ^,^ ^25^ ^)^. Those points of evidence supported the recommendation of reducing the consumption of total fat and SFA in Western nations where fat intake is higher than in other parts of the world^(^ ^26^ ^)^. The consequential nutrition transitions in developed countries led to behavioural changes that reduced diet-related NCD with the decreased intakes of refined foods, meats and dairy products and the increased intakes of fruits, vegetables and complex carbohydrates^(^ ^23^ ^)^. A study using National Health and Nutrition Examination Survey data on trends in intake of energy and macronutrients intake from 1971–1974 through 1999–2000 showed a statistically significant decrease in intake of fat in the US population conforming to the dietary guideline^(^ ^27^ ^)^. Population energy and macronutrient intakes have remained relatively stable over the decades^(^ ^28^ ^,^ ^29^ ^)^. Although the prevalence of obesity is still increasing in the USA, the rate of increase has slowed in the most recent decade to about half of what it was two decades ago (22·9 % in 1988–1994, 30·4 % in 1999–2002, 33·4 % in 2003–2006 and 36·4 % in 2011–2014)^(^ ^29^ ^)^.

Nevertheless, the general dietary recommendation on reducing fat has recently been challenged, as the inverse increase in carbohydrate intake has been suggested as a risk factor for NCD^(^ ^26^ ^,^ ^30^ ^)^. Recent reviews have indicated that there is no association between SFA intake and CHD. Further, a meta-analysis of prospective cohort studies showed no significant association of dietary SFA with an increased risk of CHD or CVD^(^ ^10^ ^)^. Several following systematic reviews of randomized controlled trials and prospective cohort studies showed no evidence for a reduction in CHD mortality with a reduction in dietary fat^(^ ^4^ ^,^ ^5^ ^)^. In the Hispanic population of Mexican origin, the percentage of energy from total fat and SFA decreased while that from carbohydrate increased between 1982–1984 and 1999–2006. Interestingly, over the same period, the prevalence of obesity and related illnesses, including type 2 diabetes, increased in the population^(^ ^31^ ^)^.

It is difficult to attribute health problems observed in a population simply to dietary changes, such as an increase in carbohydrate as a substitute for fat. The trends in energy intake of various populations show a steady increase and changes in SFA intake above the dietary guidelines parallel the changes in the prevalence of obesity and diet-related NCD^(^ ^28^ ^,^ ^29^ ^)^. The lack of any association of SFA with CVD after controlling for energy may suggest that the contributions of macronutrients are comparable to each other. Many epidemiological studies in which macronutrients were substituted for SFA found no associations between SFA and CHD risk^(^ ^10^ ^)^: these studies did not specify what macronutrients were used as substitutes for SFA and did not distinguish between simple *v*. complex carbohydrates replacing SFA^(^ ^9^ ^)^. Since different types of carbohydrate result in different metabolic effects on blood sugar levels, carbohydrate type may be a stronger indicator of CVD risk than total carbohydrate^(^ ^32^ ^,^ ^33^ ^)^. In several population studies, it is common for SFA to be replaced by refined starches and/or added sugars^(^ ^9^ ^)^. Replacing dietary SFA with other types of fat, especially *cis*-PUFA, has been reported to reduce CVD risk in several randomized controlled trials^(^ ^34^ ^,^ ^35^ ^)^ and observational studies^(^ ^36^ ^)^. According to the majority of the literature, dietary saturated fats, compared with carbohydrates and unsaturated fats, raise plasma LDL-cholesterol, a causal risk factor for CHD^(^ ^1^ ^,^ ^11^ ^,^ ^34^ ^–^ ^37^ ^)^.

In addition, individual SFA affect plasma lipoprotein levels differently^(^ ^11^ ^,^ ^37^ ^–^ ^39^ ^)^: myristic acid and palmitic acids have the greatest effect, while stearic acid has not shown any effect on elevating blood cholesterol. The most effective replacement for SFA in terms of CHD has been shown to be linoleic acid. Therefore, not only should the type of fat be considered, but also so should food sources which have specific fatty acid profiles. Pork, milk and instant noodles, which were the major source of total fat and SFA in the Korean population, have high myristic and palmitic acid contents^(^ ^15^ ^)^. Low-fat dairy and the replacement of fat sources with oils, nuts and seeds should be promoted.

Nutrition transitions have been reported worldwide along with changes in demographics and economic transitions, but these changes have been seen substantially more in developing countries^(^ ^23^ ^,^ ^40^ ^,^ ^41^ ^)^. In the present study focusing on the Korean population, we found that the energy consumption of the Korean population increased gradually along with total fat consumption and the proportion of energy from fat. The increase of total fat intake is explained by increases in all of the components proportionally from the 2007–2009 to the 2013–2015 survey period (*P*<0·05): saturated (from 4·7 to 5·3 %E), monounsaturated (4·6 to 5·8 %E) and polyunsaturated (4·2 to 4·7 %E). Traditionally, the Korean population has quite a low proportion of energy from fat. The observed nutrition transitions are the increase in consumption of SFA along with the increase in prevalence of hypercholesterolaemia. The proportion of energy from carbohydrate has traditionally been high in the Korean population with a high prevalence of hypertriacylglycerolaemia.

Therefore, public dietary recommendations need to be carefully developed based on unique situations driven by evidence on the proportion of energy from fat and carbohydrate. In order to address the emerging health problems related to dietary behaviours in specific populations, recommendations need to be specific but robust enough to consider global views of changing trends in diet and lifestyle in relation to health outcomes. Even though the Korean population consumes low energy percentage from SFA, researchers and policy makers need to be mindful of the changing trends. The types of carbohydrate and fat and their sources have to be considered simultaneously along with the total amount, in order to avoid unfavourable nutrition transitions while enabling regionally, culturally and nationally acceptable healthful behavioural changes in a timely manner.

## Conclusions

Although the intakes of total fat and SFA are relatively low in Korea compared with those in Western countries, the public health messages in Korea have been very similar to those in Western countries. The findings of the present study appear to justify the efforts to control total fat and SFA in the diet. Furthermore, the guidelines can expand on the specific food sources for complex carbohydrates and PUFA as they are desirable substitutes for SFA. Most of all, the issue of energy balance rather than the issue of low carbohydrates or of low fats should be considered, because it was also observed that unfavourable changes in chronic health conditions accompanied increased energy intake. The present study simply estimated the population-level consumption of different types of fat and the trends of the recent nine years. However, it is the first study to document population-level trends of nationally representative samples in Korea using the recently constructed comprehensive fatty acid database. This work can help shed light on public health concerns related to fat consumption and provide helpful information for setting adequate macronutrient distributions for the development of population-specific preventive strategies against diet-related illness.

## Author ORCID

Jae Eun Shim, 0000-0001-8458-9112.
